# Practical Applications of NMR to Solve Real-World Problems

**DOI:** 10.3390/molecules26237091

**Published:** 2021-11-24

**Authors:** Robert G. Brinson

**Affiliations:** Institute for Bioscience and Biotechnology Research, National Institute of Standards and Technology and the University of Maryland, 9600 Gudelsky Drive, Rockville, MD 20850, USA; robert.brinson@nist.gov

Nuclear magnetic resonance spectroscopy (NMR) is known to be a powerful technique for the characterization of small molecules and structural and dynamics studies of biomolecules. While it was once primarily used only in academia, this technique is now routinely utilized in many industry sectors, government agencies, and other applied research activities with great benefits. Such areas include agriculture, metabolomics and complex mixtures, manufacturing and process monitoring, pharmaceuticals (biologics and small molecules), national security, forensics, energy, and renewables. Many similar technical challenges, and often, regulatory burdens, are shared across these fields; yet, there is often little cross-talk between the various NMR practitioners in each of these focused areas. Greater sharing of experience and expertise amongst practitioners of applied NMR would be of benefit to all.

With this in mind, this Special Issue collected 10 original papers covering four sectors, including the Oil Industry, Nanostructured Systems and Materials, Metabolomics, and Biologics. These contributions show how this technology is now used in very disparate areas. Further, the NMR systems discussed range from low-field magnetic resonance imaging (MRI), 18.2 MHz relaxometry, and high fields up to 700 MHz. These articles show that NMR technology can be tailored for the specific needs of an industry.

*Oil Industry*: In their work entitled “*Study on Nuclear Magnetic Resonance Logging T_2_ Spectrum Shape Correction of Sandstone Reservoirs in Oil-Based Mud Wells*” Sun et al. presented a need to improve the NMR logging data in order to accurately determine the physical parameters of the surrounding sandstone reservoirs to improve drilling operations [1]. During drilling operations, overbalanced pressure causes the infiltration of oil-based drilling lubricant into the surrounding sediment, resulting in a shorter *T_2_* relaxation. The authors determined a *T_2_* correction factor so that accurate NMR logging data could be determined for the morphology of the sandstone reservoirs.

In their article entitled “*Multiphase Flow Regime Characterization and Liquid Flow Measurement Using Low-Field Magnetic Resonance Imaging*”, Tromp and Cerioni simulated a multiphase flow in a pipeline [2]. They used low-field magnetic resonance imaging (MRI) to measure various types of flow and found the MRI measurement offered an accurate flow determination that was not dependent upon the multiphasic character of the flow. They suggest that the MRI measurement technology is robust enough for routine implementation in the petroleum industry.

*Nanostructured Systems and Materials*: In an article by Shelyapina et al., entitled “*^1^H NMR Study of the HCa_2_Nb_3_O_10_ Photocatalyst with Different Hydration Levels*”, ^1^H magic angle spinning (MAS) NMR and ^1^H spin-lattice relaxation time in the rotating frame (*T_1__ρ_*) were used to investigate the hydration properties of a layered perovskite-like oxide, HCa_2_Nb_3_O_10_ [3]. This specific perovskite-like oxide is known to have photocatalytic properties, and the state of the water molecules influences these properties. From their ^1^H NMR studies, the authors determined that HCa_2_Nb_3_O_10_ has three different hydration forms: α-form HCa_2_Nb_3_O_10_·1.6H_2_O, β-form HCa_2_Nb_3_O_10_·0.8H_2_O, and γ-form HCa_2_Nb_3_O_10_·0.1H_2_O.

In an effort to improve separation and purification protocols, a new method was introduced to study the solvent separation efficiency of metal–organic frameworks (MOFs) [4]. As described by Wagemann et al. in “*Screening Metal–Organic Frameworks for Separation of Binary Solvent Mixtures by Compact NMR Relaxometry*”, the measurement of effective transverse relaxation by low-field ^1^H NMR relaxometry of a two-solvent mixture was used to establish correlation curves in relation to their mass proportion. Using the model MOF, powdered UiO-66 (Zr), the authors demonstrated great accuracy with their method and agreement with solution-state ^1^H NMR measurements. They suggest their method could be integrated into the workflow of a synthetic laboratory due to their method’s accuracy, ability for automation, and time savings.

*Metabolomics*: Honrao and coworkers presented paramagnetic relaxation enhancement (PRE) to increase sensitivity for metabolomics measurements in “*Gadolinium-Based Paramagnetic Relaxation Enhancement Agent Enhances Sensitivity for NUS Multidimensional NMR-Based Metabolomics*” [5]. By doping reference and test metabolite samples with gadolinium, the authors demonstrated a sensitivity enhancement, especially for the weakest signals, resulting in a lower limit of detection (LOD) and limit of quantification (LOQ). Further, their method maintains the linearity of intensity needed for the concentration range of metabolites tested.

The article “*Establishing a Metabolite Extraction Method to Study the Metabolome of* Blastocystis *Using NMR*” by Newton et al. describes a method of extracting metabolites from *Blastocytis* [6]. More in-depth analysis of the role of metabolism is needed to understand this pathogenic human parasite. The researchers tested a variety of extraction conditions, including solvent, lysis technique, and temperature. They optimized the first published extraction protocol for NMR analysis of the *Blastocystis* metabolome.

A method to combine both NMR and high-resolution mass spectrometry (HRMS) was proposed by Petrella et al. in “*Personalized Metabolic Profile by Synergic Use of NMR and HRMS*” [7]. Their SYNHMET (SYnergic use of NMR and HRMS for METabolomics) protocol afforded a synergistic characterization of urine metabolites, leading to higher accuracy in identification and concentration determination. In all, this new method detected 165 metabolites from patients with varying health profiles.

*Biologics*: In an article entitled “*Glycosylation States on Intact Proteins Determined by NMR Spectroscopy*”, Hargett et al. proposed an NMR method to selectively analyze the glycans from intact glycoproteins using RNase A and RNase B as model proteins [8]. Specifically, they implemented a ^1^H,^13^C HSQC-TOCSY at natural isotopic abundance. By optimizing the TOCSY mixing period, they could suppress the protein NMR signals, allowing for the analysis of the glycans independent of the protein. They plan to apply their work to polysaccharide conjugate vaccines.

The Wikström laboratory performed a comparative analysis of methods to evaluate higher-order structures (HOS) of monoclonal antibody-based therapeutics in “*Use of the 2D ^1^H-^13^C HSQC NMR Methyl Region to Evaluate the Higher Order Structural Integrity of Biopharmaceuticals*” [9]. Comparing near-ultraviolet circular dichroism (NUV-CD), intrinsic fluorescence spectroscopy (FLD), and natural abundance ^1^H,^13^C HSQC experiments, the low-resolution methods only provided limited discrimination between folded and unfolded samples of IgG1 and IgG2 subtypes. However, the NMR HOS method demonstrated high sensitivity to even subtle changes in higher-order structures (HOS) and may serve as a replacement method for low-resolution HOS assessments.

Another study, entitled “*NMR Spectroscopy for Protein Higher Order Structure Similarity Assessment in Formulated Drug Products*” by Wang et al., looked at defining similarity metrics for protein-based drug products (DPs) [10]. Using several different peptide and proteins, they presented a variety of metrics for establishing the similarity between a biosimilar DPs. Their overall goal was to establish a fit-for-purpose quantitative metric to establish similarity for a biosimilar.

As a guest editor of this Special Issue, I would like to extend my appreciation to all the authors for their excellent contributions and for their valuable support. I additionally thank the reviewers for their time and feedback.
